# Prognostic value of cutaneous reinnervation with GAP-43 in oxaliplatin-induced neuropathy

**DOI:** 10.1007/s00415-022-11035-9

**Published:** 2022-03-08

**Authors:** Merve Albayrak, Carolina Figueras, Elia Seguí, Michela Campolo, Eva Gabarrón, Reinaldo Moreno, Joan Maurel, Jordi Casanova-Molla

**Affiliations:** 1 Neurology Department, Hospital Clínic of Barcelona, Barcelona, Catalonia Spain; 2 Medical Oncology Department, Hospital Clínic of Barcelona, Barcelona, Catalonia Spain; 3 Institut d’Investigació Biomedica Agustí Pi Sunyer (IDIBAPS), Barcelona, Catalonia Spain; 4 Department of Medicine, Facultat de Medicina I Ciències de La Salut-Campus Clinic, Universitat de Barcelona, C/Casanova, 143, 08036 Barcelona, Catalonia Spain

**Keywords:** Oxaliplatin-induced neuropathy, Small fiber neuropathy, Intra-epidermal nerve fiber density, GAP-43, Re-innervation

## Abstract

**Background and purpose:**

Oxaliplatin-induced neuropathy (OIN) implies axonal damage of both small and large sensory nerve fibers. We aimed at comparing the neurophysiological changes occurred after treatment and the capability to recovery based on histological marker of re-innervation GAP-43.

**Methods:**

48 patients with cancer were assessed before and after chemotherapy (at 3 months and 12 months if available). We recorded ulnar and sural sensory nerve action potentials (SNAP), determined quantitative sensory thresholds for warm and cold (WDT, CDT), pain thresholds and collected a distal biopsy of skin to assess the intra-epidermal nerve fiber density (IENFD) with PGP9.5 and GAP-43 markers (in a subgroup of 19 patients).

**Results:**

Increased WDT and CDT as well as diminished IENFD at distal leg were already found in 30% of oncologic patients before treatment. After oxaliplatin, there was a significant increase in thermal thresholds in 52% of patients, and a decrease of SNAP amplitude in the sural nerve in 67% patients. IENFD was reduced in 47% and remained unchanged in 37% after oxiplatin. The density of GAP-43 + fibers and GAP-43/PGP 9.5 ratio was similar before and after treatment showing that cutaneous re-innervation is preserved despite no clinical recovery was observed after one year.

**Conclusion:**

Non-selective axonal loss affects sensory fibers in OIN. However, the presence of intra-epidermal regenerative sprouts detected by GAP-43 may reduce the impact of neurotoxicity in the small fibers with long-term sequelae mostly on myelinated nerve endings. Pre-oxaliplatin GAP-43 failed to identify patients with higher risk of damage or worse recovery after treatment.

## Introduction

Peripheral sensory neurons and nerve fibers are particularly vulnerable to oxaliplatin. More than sixty percent of patients at 3 months receiving oxaliplatin treatment develop a chronic sensory polyneuropathy, which is the principal side effect of this chemotherapy drug [1, 2]. Oxaliplatin-induced neuropathy (OIN) is characterized by long-lasting loss of sensation, allodynia and other forms of neuropathic pain, with moderate interference in quality of life [3]. It is still unclear whether axonal damage affects predominantly small fibers or it is a non-selective damage of large and small fibers. The interest in the characterization of such neuropathy has recently increased because of the need to find markers of risk at pre-oxaliplatin evaluation or prognostic signs after treatment.

Oxaliplatin has an acute neurotoxic effect on nerve excitability because of reversible inactivation of slow sodium channels [4, 5], but there can also be a slowly developing axonal damage with treatment progression. In fact, the cumulative dose of oxaliplatin is one of the risk factors that has been associated with OIN as well as the single-dose intensity [6], similarly to what has been reported with cisplatin [7, 8]. In a recent publication, Krøigård et al. reported a linear increase in neuropathy scores from early phases of oxaliplatin treatment that did not necessarily exceed a specific threshold on oxaliplatin dose [9]. In addition, the authors found increased vibration threshold, as the first indicator of OIN affecting large, myelinated neurons or fibers, after receiving 25% of the planned dose. In comparison, the intra-epidermal nerve density (IENFD) was not reduced, supporting the idea that sensory nerve fibers are not equally affected [9]. In fact, the changes in distal IENFD reported until now after receiving oxaliplatin show high disparity, including reduction of 20–60% [9–12], no change [13] or increase in some cases [10, 14], in comparison to baseline. Intriguing findings coming out from reviewing these articles are the reduced IENFD at baseline examination, prior to receiving chemotherapy [12, 14]. It is possible that oncologic patients have already damaged distal sensory nerves before starting the treatment. This fits with a functional deficit in warm sensation found with quantitative sensory testing (QST) in oncologic patients before starting chemotherapy in their hands [12, 15–17] and feet [9, 12, 18]. However, such evaluation (before treatment) has not been often reported making the correct interpretation of the results obtained on QST after chemotherapy difficult. This could furnish an explanation for the high variability on QST results reported until now after chemotherapy.

Restoration of IENFD was reported possible in oncologic patients after induced denervation with high-dose capsaicin patch [12]. These authors found an increase in IENFD of both, the pan-axonal marker protein gene product 9.5 (PGP 9.5) and the growth-associated protein-43 (GAP-43) immuno-reactive (-ir) fibers post patch application. However, it is unknown whether histological markers of re-innervation as the GAP-43 would help to identify patients with higher risk to develop OIN or higher capability to recovery. Thus, we conducted this study to follow up a cohort of patients before and after oxaliplatin treatment, investigating clinical, neurophysiological and histological data of changes at different sensory fibers and the relationship between intra-epidermal nerve fiber loss and its regeneration with GAP-43.

## Methods

### Patients

We prospectively enrolled between June 2016 and March 2021 at Hospital Clinic of Barcelona patients taking oxaliplatin plus leucovorin and 5-fluorouracil (FOLFOX) or oxaliplatin plus capecitabine (XELOX), either in the adjuvant or metastatic setting treatment for gastrointestinal malignancies. They were evaluated by means of physical examination, nerve conduction studies (NCS) and thermal-QST (TST). A skin punch biopsy at distal foot was done only when a patient’s full agreement was given. Patients were included in the study if they received at least three cycles of oxaliplatin. Patients were excluded if life expectancy was low or if there was evidence of pre-existing conditions other than colorectal cancer that could produce neuropathy, including diabetes (except if the diagnosis was made after being recruited), paraneoplastic syndrome or fibromyalgia. Also, we excluded patients with severe radiculopathy or myelopathy to avoid interference with our evaluation. Paraneoplastic syndrome was not suspected in any patient during the chemotherapy or follow-up and, consequently, anti-neuronal antibodies were not determined. All participants gave their written informed consent for the study, which was approved by the local Ethics Committee at the Hospital Clinic of Barcelona, Reg. HCB/2016/0689 in agreement with the World Medical Association Declaration of Helsinki.

## Procedure

We planned for at least two evaluation sessions: before-treatment (1 week before initiating oxaliplatin) and after-treatment (3 months after the last administration of oxaliplatin). In those patients that was possible, we made additionally a one year after, for long follow-up. Before-treatment and after-treatment sessions included all clinical examination, NCS, TST and skin biopsy.

### Clinical examination

We asked for sensory symptoms and collected possible sensory signs indicating neuropathy in the physical neurological examination on each session. The clinical assessment was completed in the evaluation after-treatment with the clinical part of the Total Neuropathy Score (TNSc) and the National Cancer Institute Common Toxicity Criteria for Adverse Events (NCI CTCAE V.4.0; Nervous system disorders; Peripheral sensory neuropathy), which was used as the gold standard for the classification of the degree of polyneuropathy (grade 1 “mild” and grade 2 or 3 “severe”).

### Nerve conduction studies (NCS)

To investigate large myelinated fiber function, patients underwent sensory NCS using surface recording electrodes with standard placement. Sensory nerve action potential (SNAP) amplitudes and conduction velocities of one nerve in lower extremities (sural nerve) and one nerve in the upper extremities (ulnar nerve) were measured using a KeypointNet electromyograph (Dantec, Natus Medical Inc., CA, USA). We considered abnormal a mean SNAP amplitude value of < 5 μV for the antidromic sural and < 4 μV for the orthodromic ulnar nerve according to -2 SDs of previously published normative reference values from our institution in healthy subjects which were for male and female between 60 to 80 years old a mean sural SNAP of 12.6 ± 3.8 μV and mean ulnar SNAP of 12 ± 4 μV [19]. We also calculated number of patients with reduction in SNAP amplitude after-treatment equal or higher than 25% compared to before-treatment.

### Thermal sensory testing (TST)

Thermoalgesic stimuli were applied with MSA device (SENSELab; Somedic, Sweden) using a Peltier-type contact thermode of 12.5 cm^2^. Starting temperature on the thermode was set at 32ºC and the rate of temperature increase was 1 °C/s. We determined warm, cold, heat pain, and cold pain detection thresholds (WDT, CPT, HPT, CPT), at the hand and foot dorsum with the method of limits [20]. The mean value obtained out of 5 stimuli on both sides was for WDT, CDT, CPT and HPT were recorded and determined whether they were within or outside the cut-off limits of 2 SDs below or above for the values gathered from the Z-score calculation on WDT and CDT. We considered values from an age- and gender-matched heathy subject’s sample of 24 subjects (age 58 ± 9.5 years, 13 females). Calculation was done with a mean and SD of 36.3° ± 1.7 to WDT and 29.7° ± 1.2 to CDT at dorsum of the foot; 33.8° ± 0.7 and 30.5° ± 0.6 respectively for WDT and CDT at dorsum of the hand. Regarding CPT and HPT, we used 20.3° ± 4 and 43.8° ± 2.4 respectively, for the foot.

### Skin biopsy and IENFD

All skin specimens were taken with a disposable 3-mm circular punch under a sterile technique from the lateral side of the distal leg, 10 cm above the lateral malleolus. In one patient, samples were obtained at the lateral side of the fourth finger in the hands to compare findings in glabrous skin regarding Meissner’s corpuscles. The tissue fixation was done with 4% paraformaldehyde over 30 min and, after being cryo-protected in sucrose 10% for 24 h, specimens were cut on sliding microtome at 50 µm. Fluorescent immunohistochemistry was performed using the free-floating technique to each skin section. Three non-consecutive sections were incubated for 24 h at room temperature with the primary antibody PGP 9.5 (panaxonal marker protein gene product 9.5 made in mouse: Ref. E3344; Amsbio, Ltd.; diluted: 1:800; Abcam, Ref. ab15503; diluted: 1:800) and GAP-43 made in rabbit (NovusBio; Ref. NB300-143; diluted 1:500). As the secondary antibody, Alexa Fluor 488 and Alexa Fluor 555 were used for double staining (Invitrogen. ThermoFisher Scientific Inc.). All slides were coded to ensure blinded data analyses. The intra-epidermal nerve fibers (IENF) count was done according to the European guidelines [21] by one trained observer (JCM) as previously published [22]. Results were compared with those of 24 remaining skin sections from controls (12 healthy subjects, 6 women and 6 men with a mean age of 53.2 ± 8.5 years).

### Data reduction, measurements, and statistical analysis

Analyses were performed using SPSS 24 with statistical significance set at *p* < 0.05. Descriptive statistics were generated for all variables. The paired *t* test was used to compare data between the before-treatment and after-treatment sessions. Values of SNAP amplitude and TST were Z-transformed based on previously reported mean and SD in healthy subjects. An independent sample *t* test was used to compare means of the tests between patient groups with severe and mild neuropathy. ANOVA with Bonferroni post hoc analysis was used to compare IENFD values for GAP-43, PGP 9.5 and GAP-43/PGP9.5 ratio among controls, pre- and post-oxaliplatin. The Spearman’s correlation was used to explore association between the clinical scales (TNSc and NCI CTCAE) and other variables with age, whereas Pearson’s correlation was used to observe association between TST, NCS, IENFD and oxaliplatin dose.

## Results

Forty-eight patients were included for this study. Table 1 shows demographic, tumor and clinical data in patients. As seen in Table 2, of the 48 patients included, 19 accepted the skin biopsy before- and after-treatment. At first session (before-treatment), no patient had any symptom or sign suggesting polyneuropathy. Most of them (94%) had sensory complaints immediately after the treatment, mainly reported as distal loss of sensation at tip of the fingers in hands and feet (94%) which was reported painful in 17%. Cold hyperalgesia was reported by 7 patients (14.6%) and we found loss of ankle tendon jerks in the majority (96%).

**Table 1:** Characteristics of patients and clinical scales

Number of patients	48
Age, mean (range)	62.7 (41–80)
Gender (%)	26 Male (54)
Patient cancer Types (%)	Colorectal (88); Gastric (6); Pancreatic (3); Esophagus (3)
Grade (%)	IIIA (18), IIIB (45), IIIC (8), IV (29)
Dose mean, (range)	1112 (528–2351)
Number of cycles (median)	4–16 (9)
Symptoms (%)	Sensory loss (95); Pain (17); Cold Hyperalgesia (15)
Neuropathy scoring	
TNSc (range)	5.9 (1–13)
NCI-CTCAE grades (*n*, %)	grade 0 (2, 4); grade 1 (29, 60); grade 2 (16, 33); grade 3 (1, 2)

*TNSc* total neuropathy score, clinical, *NCI CTCAE* common terminology criteria for adverse events from the National Cancer Institute

**Table 2:** Neurophysiological results (NCS and TST) before and after oxaliplatin

NCS	Reference values in healthy subjects	Patients treated with oxaliplatin		
		Pre	Post	*p *value
Sural	12.6 ± 3.8	13.9 ± 7.3	9.8 ± 7.1	< *0.001*
Ulnar	12.0 ± 4.0	8.3 ± 3.5	5.9 ± 4.0	< *0.001*
TST				
WDT foot	36.3 ± 1.7	38.4 ± 3.1	40.8 ± 3.8	< *0.001*
CDT foot	29.7 ± 1.2	27.8 ± 2.9	26.1 ± 4.3	*0.06*
WDT hand	33.8 ± 0.7	35.0 ± 1.4	35.9 ± 2.8	*0.04*
CDT hand	30.5 ± 0.6	29.1 ± 1.6	29.1 ± 3.1	> *0.05*
Cold pain threshold	15.0 ± 5.0	12.7 ± 4.8	16.0 ± 6.5	*0.003*
Heat pain threshold	43.8 ± 2.4	43.7 ± 3.3	44.3 ± 3.7	> 0.05

Mean values and SDs over time in patients and healthy controls. *NCS* Nerve conduction studies (amplitude of SNAP), *TST* thermal sensory testing (WDT: warm detection threshold; CDT: cold detection threshold). Statistical comparison was done with *t* test paired between results pre (before treatment) and post (3 months after treatment). Cold and Heat pain thresholds have been reported at dorsum of the foot. Values in healthy subjects have been added in the table as reference

## NCS

**Fig. 1:**
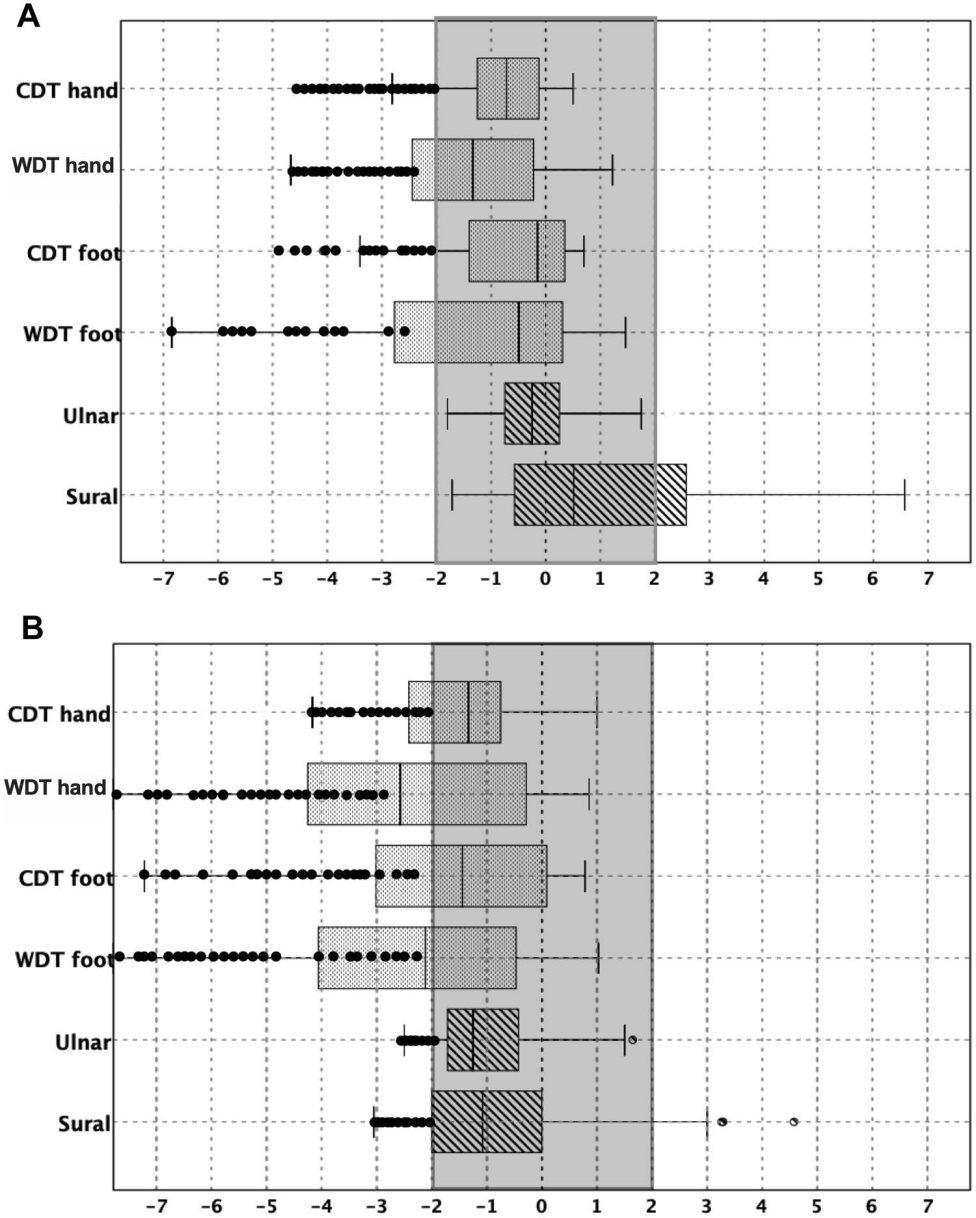
Z-scores corresponding to NCS and TST. Legend: **A** before-oxaliplatin and **B** 3 months after last oxaliplatin cycle. Results of nerve conduction studies (SNAP amplitude for sural and ulnar nerve) and thermic sensory testing (CS: cold sensation; WS warm sensation) at foot and hands. The limits corresponding to 2 SD are shown as gray area. Black dots represent subjects out of normality limits

The results on NCS at the before-treatment examination showed that all patients had normal SNAP amplitude values respectively for sural (mean 15.$5$±7 μV) and ulnar (mean 8.2 ± 4 μV) nerves as well as conduction velocity (mean 44 ± 16 m/s to both nerves). Figure 1A shows these results as a Z-score representation before starting chemotherapy.

The amplitudes of sural and ulnar SNAPs in the after-treatment evaluation were significantly reduced (*p* < 0.01) with respect to those in the before-treatment examination. A reduction in the sural SNAP amplitude equal or higher than 25% after-treatment resulted in 32 patients (67%) and it was of 24 patients (50%) in the ulnar nerve. The average SNAP amplitude loss was in sural nerve of − 46.4 ± 23.6% and in ulnar nerve − 55.2 ± 20.3% without statistically significant difference between both nerves. In addition, we calculated the number of patients whose sural and ulnar SNAP amplitudes were below the cutoff normal limits after-treatment which was in 11 patients (23%) for sural and in 18 patients (38%) for ulnar nerves. These Z-score abnormalities are showed in Fig. 1B and mean values with SD are provided in Table 2. Statistically significant correlations were found between the reduction of SNAP amplitude values and the TNSc and NCI CTCAE, (*p* = 0.03 *r*_s_ = 0.3 and *p* = 0.005 *r*_s_ = 0.4, respectively) at the examination after-treatment.

### TST

Abnormal values were observed in the before-treatment evaluation for WDT at foot in 13 patients (27%) and at hand in 14 patients (29%); for CDT, we found abnormal results at foot in 14 patients (29%) and at hand in 22 patients (45%). It was coincident, both abnormal at foot for WDT and CDT in only 7 (15%) patients. Figure 1A shows the results of the Z-score transformation. The number of patients showing TST abnormalities increased when evaluated 3 months after oxaliplatin (Fig. 1B). After finishing the treatment, the mean values for WDT and CDT were out of normal limits in 25 patients (52%) for foot WDT and 22 patients (45%) for foot CDT. It was abnormal also in 25 patients (52%) for hand WDT and 16 patients (33%) for hand CDT. Cold hyperalgesia was noted in 14 patients (29%), who reported CPT at higher temperatures than in the before-treatment evaluation. No statistically significant differences were found between data on HPT at hand and foot. Results are provided as mean and standard deviation in Table 2 with the statistical comparison.

### Cutaneous innervation and re-innervation

**Fig. 2:**
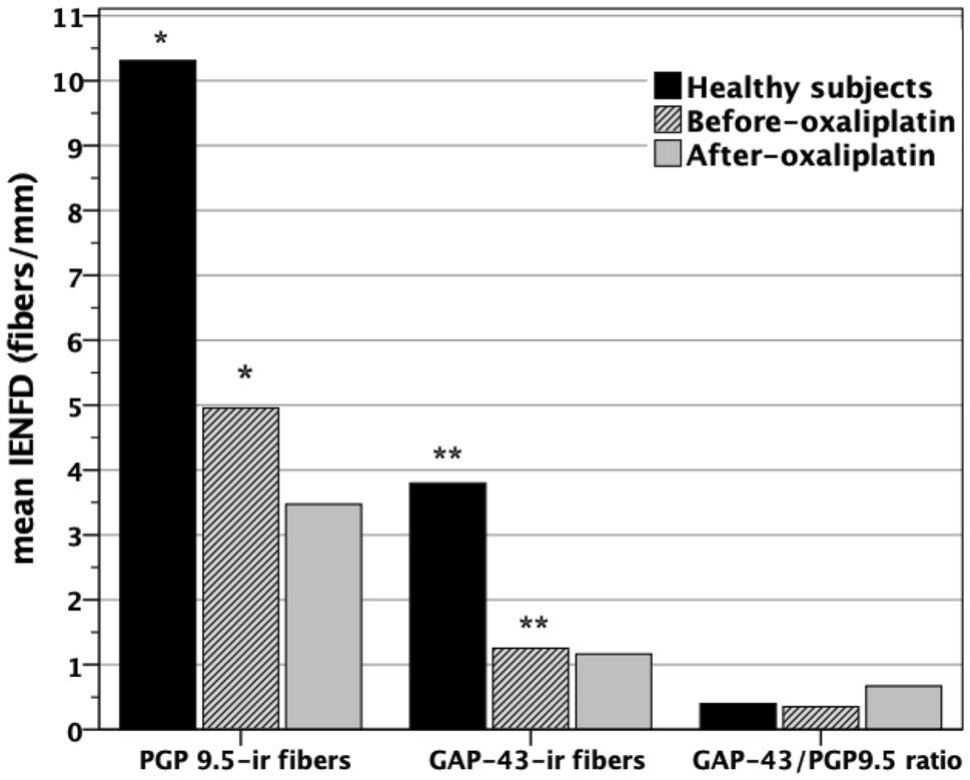
Study of skin innervation with PGP 9.5 and GAP-43. Mean intra-epidermal nerve fiber density (IENFD) at distal leg in healthy subjects, before-oxaliplatin and 3 months after last oxaliplatin cycle. Statistically significant differences between healthy subjects and before-oxaliplatin are indicated for PGP 9.5 (*) and GAP-43 (**). The PGP 9.5/GAP-43 ratio (%) is included also for comparison in the same graph

**Fig. 3:**
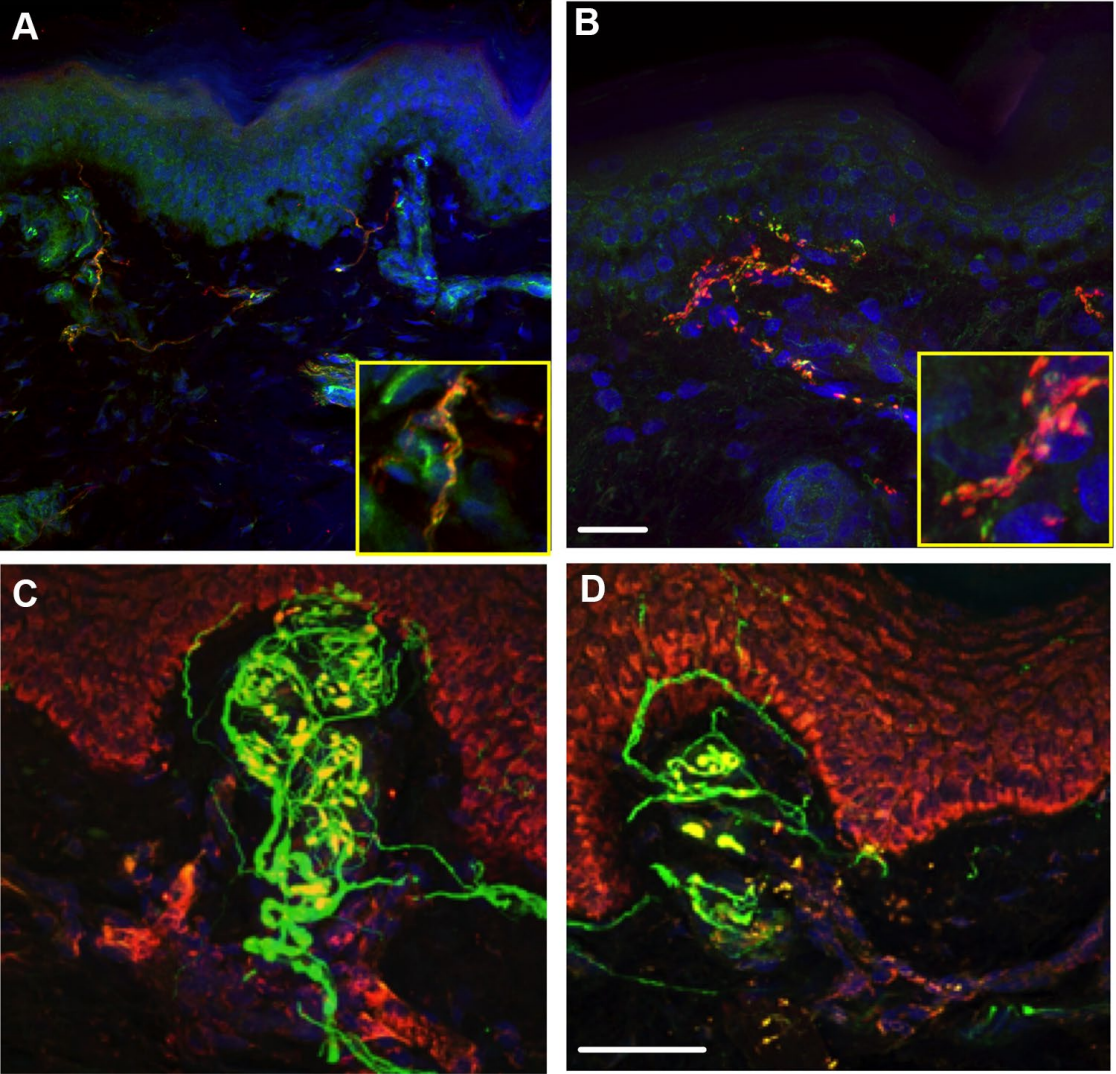
Examples of double immunofluorescence labeling images with confocal microscope. **A**, **B** Double-staining with PGP 9.5 (red) and GAP-43 (green) from one patient before (**A**) and after (**B**) receiving oxaliplatin. In image **A**, it is possible to identify co-localization between both PGP 9.5 and GAP-43 (inset) as well as no-coincident nerve fibers (GAP-43 +); Despite generalized axonal loss in image **B**, it is possible to find PGP 9.5 and GAP-43 + remaining dermal fibers; Image shows generalized fragmentations of dermal fibers. **C**, **D** Double-staining of PGP 9.5 and OXPHOS (keratinocytes) with DAPI (nucleus) in a skin biopsy from 4th lateral aspect of finger at hand (glabrous skin) showing a Meissner corpuscle before (**C)** and after (**D**) receiving oxaliplatin. Scale bar of 50 µm is placed as reference in right images

A total of 19 patients (40% of all patients recruited) gave permission to perform a distal skin biopsy before and after 3 months of oxaliplatin treatment. Mean data on IENFD are presented in Fig. 2 for samples from control subjects and those 19 patients. Patients had statistically significant lower IENFD at the before-treatment evaluation, with 6 patients (32%) showing lower IENFD than our reference cut-off values [22]. In after-oxaliplatin, we found abnormal low IENFD in all patients (100%). However, not all individual changes after receiving oxaliplatin went in the same direction (Table 3): a decrease was seen in 9 patients (47%), no changes were seen in 7 patients (37%) and an increase in fiber density was seen in 3 patients (16%). From morphological analysis, most of dermal nerve fibers showed the presence of axonal swellings and fragmentations. An example of microscopy images is shown at Fig. 3.

**Table 3:** Clinical, neurophysiological and histopathological data in patients before and after oxaliplatin

	Age years /sex	OXA dose/cycles	TNSc	Sural SNAP (amplitude)		WDT (°C)		CDT (°C)		IENFD PGP 9.5 (fibers/mm)		IENFD GAP43 (fibers/mm)		GAP/PGP ratio	
				Pre	Post	Pre	Post	Pre	Post	Pre	Post	Pre	Post	Pre	Post
1	72/F	704/10	3	26	21	39.7*	39.1*	27.0*	29	10.0	3.0↓	4.1	2.0↓	0.4	0.7**↑**
2	57/M	1152/11	4	11	6*	41.0*	44.8*	30.1	29.9	2.4*	0.3↓	0.7	0.7	0.3	2.3**↑**
3	69/M	1505/6	8	9	4*	37.6	45.5*	30.1	26.6*	4.3*	6.0 **↑**	0.0	0.5 **↑**	0.0	0.1
4	46/F	810/6	10	26	12*	35.3	35.7	30.6	29.8	12.5	0.7↓	1.4	1.1	0.1	1.6**↑**
5	60/M	1014/11	5	8	6	35.9	44.0*	29.8	26.8*	5.8*	5.6	2.0	0.8↓	0.3	0.1
6	69/M	840/12	9	6	2*	35.7	37.1	30.0	29.5	2.4*	3.4	1.1	1.5	0.5	0.5
7	44/M	750/12	6	11	8*	36.0	42.3*	30.1	29.8	10.7	8.5↓	2.3	1.5↓	0.2	0.2
8	67/F	840/8	4	11	9	35.8	36.4	29.6	29.7	4.3*	6.6**↑**	1.5	2.0 **↑**	0.4	0.3
9	64/M	775/6	9	15	2*	39.9*	43.1*	29.8	25.3*	5.2*	2.1↓	1.0	0.0↓	0.2	0.0
10	65/F	924/6	0	13	14	47.0*	41.9*	23.2*	26.4*	7.5*	6.0↓	1.3	1.0	0.2	0.2
11	61/M	2100/12	9	11	4*	38.6*	43.8*	29.3	30	5.0*	2.8↓	1.6	2.3 **↑**	0.3	0.8
12	61/M	942/8	13	20	1*	38.4*	41.0*	30.3	30	3.4*	6.0**↑**	1.4	1.3	0.4	0.2
13	66/M	1402/16	10	12	1*	35.6	35.6	30.0	30.6	10.1	6.0↓	0.0	0.0	0.0	0.0
14	60/F	660/8	9	15	15	37.2	39.0*	30.3	29	2.0*	1.8	1.0	1.4	0.5	0.8
15	59/M	1378/12	10	15	10*	44.0*	43.6*	25.5*	25*	0.7*	0.3	0.7	0.4	1.0	1.3
16	56/M	1281/6	6	15	7*	36.0	49.6*	29.0	10*	1.9*	2.3	1.0	0.8	0.5	0.4
17	61/F	1071/9	4	24	24	36.2	37.0	29.1	27.7	1.9*	1.8	0.0	0.0	0.0	0.0
18	69/M	1428/9	10	8	2*	36.0	43.4*	29.0	23.6*	2.0*	1.4	0.8	1.8 **↑**	0.4	1.3 **↑**
19	41/F	771/6	3	26	25	36.0	36.7	29.3	24.6*	2.0*	1.4↓	2.1	2.9 **↑**	1.0	2.1 **↑**

Thermal sensory testing: WDT (warm detection threshold) and CDT (cold detection threshold) were tested at dorsum of the foot; IENFD (intra-epidermal nerve fiber density) was calculated as mean of three non-correlative skin sections obtained from distal skin biopsy (10 cm above ankle); Asterisks (*) mean abnormal results based on 25% reduction of sural SNAP amplitude and 2 SD of WDT (39 °C) and CDT (27.5 °C). Arrows (**↑** or↓) indicate significant changes considered if > 0.5 unit

Regarding markers of re-innervation, GAP 43-ir IENF were counted always lower (20 to 35%) than those visualized to PGP 9.5 in samples from healthy controls and patients, with GAP-43/PGP 9.5 ratio of 0.4 ± 1.7 in control samples and 0.36 ± 0.3 in patients before-treatment. The change observed in IENFD related to oxaliplatin exposure was not significantly different for GAP-43 than for PGP 9.5 (Fig. 2). Absence of change occurred in 10 patients (53%) and an increase of GAP 43-ir IENF was seen in 5 patients (26%). The calculated GAP-43/PGP9.5 ratio showed an increment after oxaliplatin in 5 patients (26%), albeit this difference was not statistically significant (Fig. 2). At sub-epidermal and deep dermal fibers, we do not appreciate qualitatively differences related to IENF, both dermal and IENF were present or decreased according to changes observed in IENF.

## Comparison between TST and IENFD data

We found 6 out of 19 patients (32%) who had abnormal WDT at foot before-treatment and reduced IENFD, which resulted of 4.0 ± 2.4 fibers/mm for PGP 9.5 and to GAP 43 marker it was 1.1 ± 0.4 fibers/mm (GAP-43/PGP 9.5 ratio of 0.4). In all except one of those patients, we found in after-oxaliplatin a decreased IENFD. Among the patients who had normal TST values in before-treatment, we had 9 that showed reduced mean IENFD of 3.0 ± 1.5 fibers/mm with PGP 9.5 and 1.1 ± 0.7 with GAP-43 (GAP-43/PGP 9.5 ratio of 0.4). None of these comparisons and correlations between these values reached statistical significance (*p* values ranging between 0.3 and 0.8). No significant correlation was found between GAP-43 density and other parameters: clinical (pain sensation), functional or IENFD at the baseline or in after-treatment.

## Relationship between large and small fiber damage

We explored the possible correlation between sural SNAP amplitude, which was considered an index of large fiber dysfunction, and data on WDT or CDT at foot, which were considered measures of small fiber dysfunction. After excluding the patients with abnormal results on TST in the before-treatment evaluation, we found 11 patients (23%) that showed signs of alterations in large and small fibers likely to oxaliplatin. There were 7 patients (15%) with signs exclusively of SFN, while 21 patients (44%) showed signs of alterations limited to large fibers. No differences in age (62 vs 61 years), dose (1174 ± 371 vs 1169 ± 336) or TNSc (5.4 ± 3.2 vs 5.7 ± 3.1) were found between patients with large fibers or small fibers alterations. Only 9 patients (19%) had NCS and TST within normal values in the after-treatment examination. The changes in abnormality rate were significant for SNAP amplitude of sural and ulnar nerves (*p* = 0.00; *r*_s_ = − 0.28, 0.36) and WDT for foot (*p* = 0.01, *r*_s_ = − 0.36), but no significant correlations were found between these values. Also, no significant correlation was found between sural SNAP amplitude and IENFD. Considering two groups of our patients based on clinical NCI CTCEA scale, mild neuropathy (grade 1) in contrast to severe (grade 2 and 3), no statistically significant differences were found for neurophysiological (SNAP amplitude), psychophysical (TST abnormalities) or histological (IENFD-ir PGP 9.5 or GAP-43 fibers) data.

## Chronic neuropathy one year after oxaliplatin

**Fig. 4:**
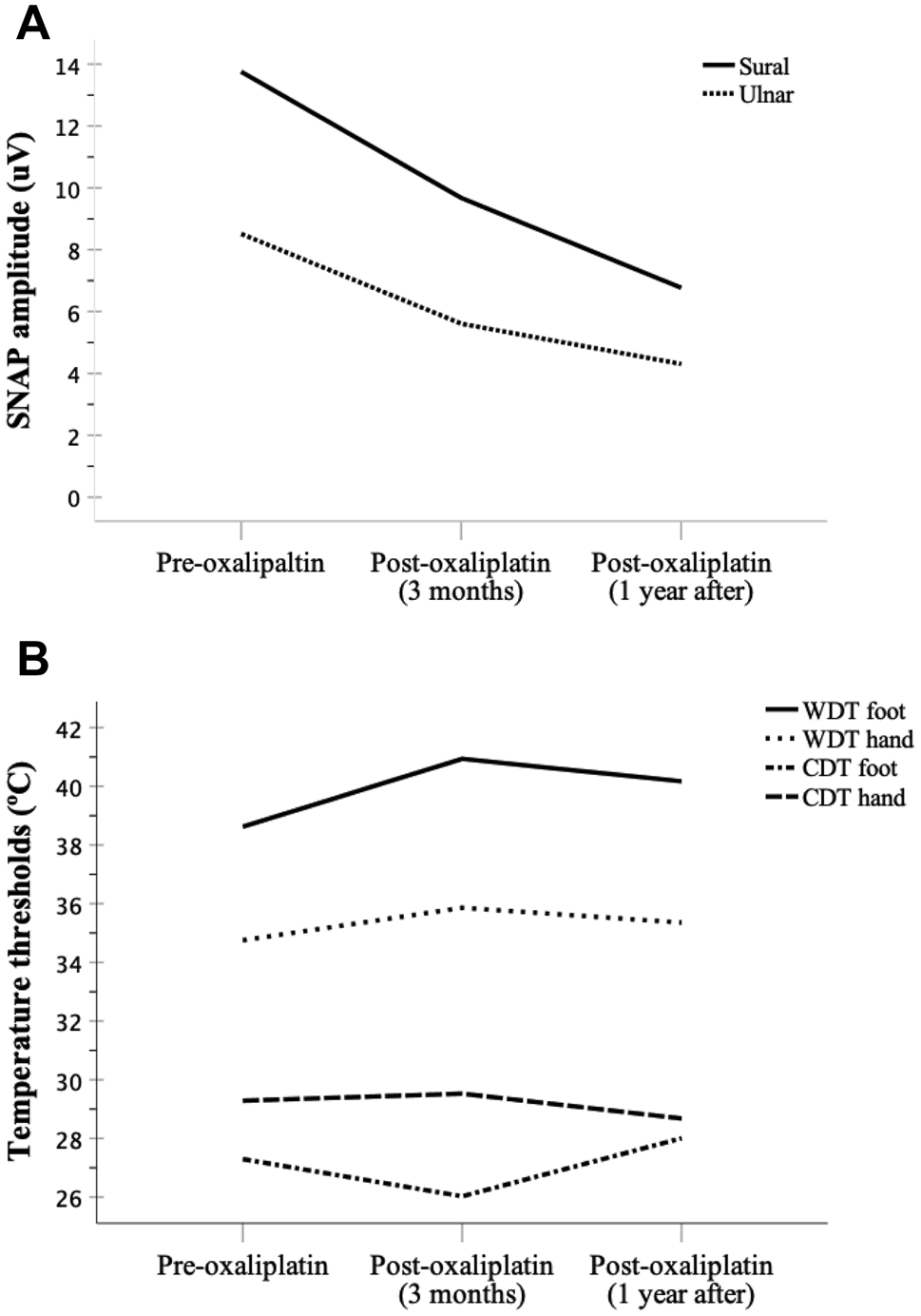
Time profile reflecting the change in NCS and TST from baseline to one-year follow-up. The line chart shows mean values of SNAP amplitude (**A**) and temperature (**B**) for warm and cold thresholds at foot and hand corresponding to the patients who were followed one year after last oxaliplatin cycle of chemotherapy. Amplitudes are expressed in microvolts and temperature in Celsius degrees

Nineteen of the 48 patients attended the 1-year last visit after chemotherapy. Mean values of SNAP amplitude one year after oxaliplatin were 6.8 ± 5 μV for sural and 4.3 ± 4 μV for ulnar nerve. Figure 4 shows the comparison of mean values at 3 months versus those obtained at 1-year follow-up. Difference resulted statistically significant for sural (9.8 ± 7.1 μV vs. 6.8 ± 5 μV, p < 0.001) and ulnar nerve (5.9 ± 4 μV vs. 4.3 ± 4 μV, *p* < 0.001). We confirmed that all those patients experienced little or no change in sensory complaints after one year of follow-up. Data obtained on WDT and CDT at foot and hand in the group of these 19 patients showed no significant change with respect to data obtained at the 3 months in after-treatment examination.

## Discussion

This study was performed as a long-term clinical, neurophysiological and histological prospective investigation in patients who received oxaliplatin. We reported an incidence of 60% mild and 35% moderate sensory neuropathy, as measured by the NCI CTCAE scale, which is in fitting with results reported in previous publications [1–3]. Our patients predominantly experienced sensory loss in a length-dependent pattern affecting both, upper and lower extremities with low incidence of neuropathic pain (17% of patients) compared to other publications (21% in reference 3). The combination of data from electro-diagnostic (NCS), psychophysical (TST) and histological (IENFD) tests before and after treatment contributes to improve characterization of OIN and has brought up evidence of a mixed involvement of large and small sensory nerve fibers with poor relevance of the cutaneous regenerating nerve sprouts as prognostic indicator for recovery.

First, we found a significant decrease in sural SNAP amplitude in 67% of our patients in after-treatment, a percentage higher than when we applied only normative cutoff value (23%) on the same results. This makes differences on NCS more pronounced when compared before and after treatment in the same patients than if only we examined patients when became symptomatic. In addition, there was a significant increase in WDT and decrease of CDT at the foot (52% and 45%, respectively) observed three months after finishing treatment. A change that was similar at the hands (52% for WDT) and less marked for CDT (33%) in our patients when compared to before-treatment results. This is in agreement to findings by different authors. It has been reported abnormal WDT and CDT at the hands [23] or at the feet [3]; others only reported low CDT at the hands [24], at the feet [25] or both, the hands and feet [26] with increased vibration detection threshold [11], despite few authors failed also to find significant thermal deficits after chemotherapy [12, 27]. Another sign described here is the loss of ankle tendon jerks, which reflect the involvement of large fibers, probably distally, at muscle spindle afferents. Moreover, with being more sensitive with distal NCS evaluating the lateral branch of the sural nerve, the dorsal sural nerve [6, 28]. In addition, we showed in one patient clear changes in myelinated distal nerve fibers in after-oxaliplatin producing the loss of PGP 9.5-ir fibers at Meissner’s corpuscles from glabrous skin taken at lateral part of fourth finger at hand (see Fig. 3). This fits with a previous report of an early decrease in distal vibration threshold [9, 26] and a greater loss of function in large fibers at the end of oxaliplatin treatment, probably all related to damage in large DRG neurons [29–31].

It is important to remark that thermal evaluation (TST) should be taken cautiously because approximately 30% of patients may present with abnormal thermal detection thresholds previous to start oxaliplatin treatment. Indeed, the possibility of a cancer-related subclinical sensory small fiber neuropathy has been suggested [16, 18, 32]. We found diminished IENFD in 6 out of 19 patients (32%) with also had abnormal WDT at baseline. Despite psychological factors, such anxiety or disturbed attention might have interfered with the reliability of testing perception previous to starting chemotherapy. The presence of abnormalities in TST before starting chemotherapy as well as significant low IENFD calls for the need for such baseline assessment to ensure a correct interpretation of the results during or after chemotherapy. However, further research with larger population of oncologic patients will be necessary because of our mean IENFD at pre-oxaliplatin evaluation resulted significantly reduced in comparison with controls (see Fig. 2). Regarding pain thresholds, we found 30% of patients with cold allodynia in the after-treatment examination even when only 14% of them reported hypersensitivity to cold. Thus, its presence is not only restricted to the acute neurotoxicity of oxaliplatin, but also persistent over time in OIN; no statistically significant difference was found between HPT before and after treatment in our patients, despite heat hyperalgesia has been reported previously as early indicator of OIN [26].

Second, the IENFD at distal leg was found more preserved than expected. No significant change was found for IENFD before and after treatment in 37% of patients as reported before by Velasco [13]. Furthermore, 3 patients showed an increase of IENFD with no clinical or functional differences in NCS or TST. This observation was made before and reported in one patient by Burakgazi [10] and in seven patients by Koskinen [14]. The high variability in IENFD findings after treatment might be explained by differences in the neurotoxic effect produced by oxaliplatin. This is not uniform in all patients because of inter-individual differences in the administered dose, levels of neuronal susceptibility or different timing of the evaluation with respect to the maximum axonal loss. On the other hand, we were able to identify the presence of nerve sprouts GAP-43-ir fibers before and after oxaliplatin exposure which indicate that small fibers keep their capacity for being regenerated. We think, therefore, that this may explain why the IENFD and dermal nerve fibers are partially preserved after oxaliplatin treatment. Actually, an increase in the GAP-43/PGP9.5 ratio has been reported in diabetic patients with cutaneous nerve fiber loss that particularly had neuropathic pain [33–35]. In our study, we found that most of the patients keep similar ratio before and after treatment but increased in 5 patients (mean GAP-43/PGP9.5 ratio of 1.6%). This change in GAP-43 was not related to any particular painful sensation or the intensity of pain. A possible explanation might be related to the non-selective neurotoxicity involving large and small fibers in OIN. In view of our findings, it comes clear that skin biopsies used to reveal re-innervation with GAP-43 have a limited role to predict a satisfactory recovery. Thus, persistent damage in myelinated fibers which seems less recoverable, might be probably the cause of chronic sensory disturbing symptoms.

A limitation of our study is the low number of skin biopsies, which relates to a lower rate of agreement of cancer patients to the procedure, especially at before-treatment visit. Another limitation is that we preferred to schedule the evaluation of patients at similar times after the end of the treatment, despite the possibility that inter-individual differences in oxaliplatin dose or in the number of cycles received could influence the uniformity of results.

In conclusion, NCS showed higher percentage of abnormal results than TST or both after oxaliplatin treatment. Deterioration of larger fibers tends to be more persistent, explaining long-term clinical findings. Skin biopsy at distal foot sites does not provide clear data regarding prediction of persistent neuropathy or recovery based on the re-innervation marker GAP-43.
